# Regulatory effects of *Lycium Barbarum* polysaccharides on immune function and their application prospects

**DOI:** 10.3389/fimmu.2025.1671971

**Published:** 2025-09-19

**Authors:** Dan Li, Jing Jin

**Affiliations:** ^1^ School of Pharmacy, Qilu Medical University, Zibo, Shandong, China; ^2^ Jining NO.1 People’s Hospital, Jining, Shandong, China

**Keywords:** *Lycium barbarum* polysaccharides, immunomodulation, anti-inflammatory activity, molecular mechanisms, functional foods

## Abstract

*Lycium barbarum*, as a traditional medicinal plant, contains abundant bioactive components, particularly *Lycium barbarum* polysaccharides (LBP), which demonstrate broad application prospects in immunomodulation, anti-aging, antioxidant, antitumor, and anti-inflammatory activities. Recent years have witnessed significant progress in research on LBP’s immunomodulatory effects, demonstrating its capacity to enhance immune function through regulating immune cell activities and suppressing inflammatory responses. LBP also exhibits notable preventive and therapeutic effects against various immune-related diseases including rheumatoid arthritis, hepatic encephalopathy, and diabetic nephropathy. Furthermore, as a natural prebiotic, LBP could modulate gut microbiota composition, improve intestinal health, and consequently exert systemic immunoenhancing effects. Despite its tremendous potential in disease prevention and treatment, LBP still faces certain limitations, particularly in extraction technologies and clinical research. This review summarizes recent advances in LBP’s immunomodulatory research, with emphasis on its mechanisms of action, reveals its potential value and application prospects in immune regulation, and provides recommendations for future research and development.

## 1 Introduction

*Lycium barbarum*, belonging to the *Solanaceae* family, has been utilized as a medicinal plant in China for over 2000 years. The *Shen Nong Ben Cao Jing* classified it as a “superior-grade” herb, attributing to it the functions of “nourishing liver and kidney, replenishing qi, strengthening essence, and promoting longevity”. In recent years, global research on *Lycium barbarum* has increased exponentially, particularly in the field of immunomodulation, which has emerged as a focal research area. The bioactivities of *Lycium barbarum* are closely associated with its diverse chemical composition. The study has revealed that *Lycium barbarum* contains various chemical components, including *Lycium barbarum* polysaccharides (LBP), carotenoids, flavonoids, phenolic compounds, amino acids, and trace elements (e.g., zinc and selenium) (1). LBP is recognized as the most important bioactive constituent, demonstrating multiple biological functions including immunomodulation, anti-inflammation, antioxidation, and anti-aging (2). As one of the most abundant and extensively studied active components, LBP features complex branched structures formed by various monosaccharides (e.g., rhamnose, fucose, arabinose, galactose, and galacturonic acid) linked via glycosidic bonds, with molecular weight (MW) typically ranging from 10 to 2300 kDa (2). Although slight variations exist in the glycosyl composition of LBP among different *Lycium barbarum* varieties, it maintains significant bioactivity in immunoregulation, antioxidation, and energy metabolism modulation.

In this narrative review, we comprehensively searched PubMed, Web of Science, and CNKI using “*Lycium barbarum* polysaccharides” and “immune” as keywords, screened and collected literature published within the last five years (2020-2025), and summarized the mechanisms underlying immunomodulatory effects of LBP. We initially retrieved a total of 247 references (22 from PubMed, 127 from CNKI, and 98 from Web of Science). After removing 17 duplicate records and excluding 66 review articles, 181 potentially eligible studies remained. Further screening of titles and abstracts led to the exclusion of 82 non-immune-related studies. Finally, we identified 99 articles focusing on the immunomodulatory effects of LBP. Through a narrative synthesis of recent advances in LBP research, we critically analyzed its mechanisms of action, elucidated its potential value in immunoregulation, and provided recommendations for future research and development.

## 2 Effects of LBP on immune cells and immune responses

### 2.1 Regulation of immune cells

#### 2.1.1 Activation of macrophages

**Figure 1:**
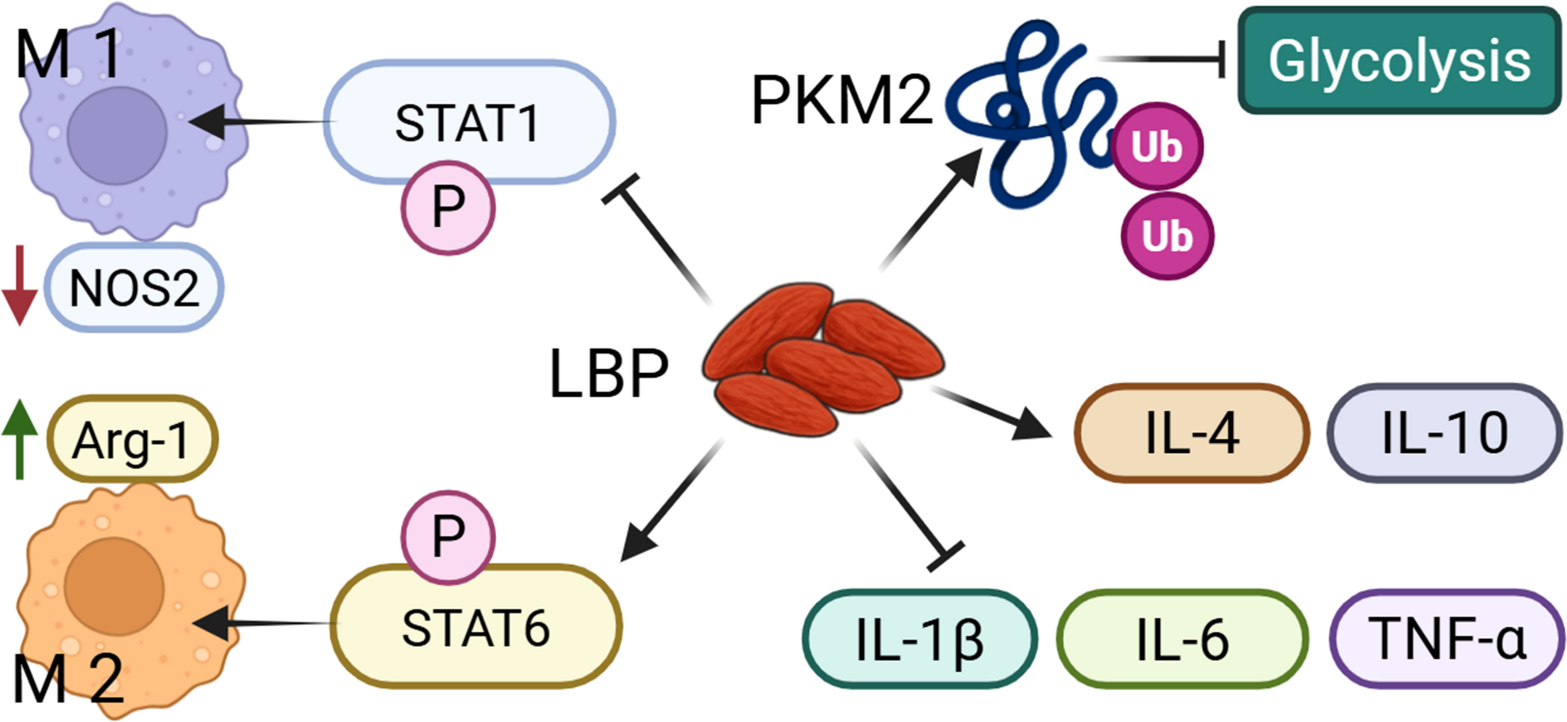
LBP could regulate the M1 and M2 polarization of macrophages. LBP promotes the polarization of macrophages toward the M1 and M2 phenotypes by inhibiting STAT1 phosphorylation and activating STAT6 phosphorylation, respectively. In addition, LBP suppresses glycolysis via PKM2 ubiquitination, and increases the production of anti-inflammatory cytokines while reduces pro-inflammatory mediators.

Macrophages are central components of the innate immune system and belong to the mononuclear phagocyte system, originating from monocytes in the bloodstream. They are widely distributed across tissues and exhibit strong phagocytic activity and immune regulatory functions, playing essential roles in infection defense, clearance of apoptotic cells, tissue repair, and inflammation modulation (3). The study has shown that LBP could regulate macrophages. In a dextran sulfate sodium (DSS)-induced colitis model mice, LBP (*intragastrica (i.g.)*, 200 mg/kg) could suppress the expression of the M1 macrophage marker nitric oxide synthase 2 (NOS2) while promoting the expression of the M2 marker arginase 1 (Arg-1). Mechanistically, LBP reduces NOS2 level by inhibiting signal transducer and activator of transcription 1 (STAT1) phosphorylation and enhances Arg-1 level by promoting STAT6 phosphorylation. This study reveals that LBP modulates macrophage polarization via the STAT1/STAT6 signaling pathway (4). Additionally, research has indicated that LBP counteracts lipopolysaccharide (LPS)-induced inflammation by modulating glycolysis and macrophage polarization. LBP (50 μg/mL) promotes the ubiquitin-mediated degradation of pyruvate kinase muscle isozyme M2 (PKM2) protein in murine RAW264.7 macrophages treated with LPS, thereby inhibiting the activity of this key glycolytic enzyme. The results in reduced lactate production and glucose consumption, as well as decreased secretion of pro-inflammatory cytokines interleukin-1 beta (IL-1β), tumor necrosis factor alpha (TNF-α), and high mobility group box 1 (HMGB1). LBP inhibits lipopolysaccharide-induced CD86 (M1 marker) increase and promotes CD206 (M2 marker) level, concurrently restoring the expression of anti-inflammatory cytokines interleukin-4 (IL-4) and interleukin-10 (IL-10) (5) (**Figure 1**). Another study reported that LBP with a MW of 27.7 kDa, obtained through water extraction and alcohol precipitation, could enhance the activity of RAW264.7 macrophages at dose of 400 and 800 μg/mL, induces cell polarization (evidenced by morphological changes and increased CD86+/CD206+ markers), and bidirectionally regulates the secretion of inflammatory cytokines TNF-α and interleukin-6 (IL-6), while suppressing excessive LPS-induced inflammatory responses (6). These findings elucidate the molecular mechanisms by which LBP exerts immunomodulatory effects through the regulation of macrophage function. Moreover, LBP (obtained by water extraction and alcohol precipitation, concentration: 400 and 800 μg/mL) reduces the secretion of pro-inflammatory cytokines (TNF-α, IL-1β, IL-6) and nitric oxide (NO) in LPS-induced RAW264.7 macrophage model (7).

#### 2.1.2 Regulation of T cell function

T cells are the key effector cells of the adaptive immune system. They mature in the thymus and possess both antigen-specific recognition and immunological memory capabilities. They play a central role in combating infections, tumor surveillance, and immune regulation by directly killing infected cells or orchestrating other immune responses (8). The study has shown that LBP (*i.g.*, 0.3 and 0.6 g/kg) alleviates skin lesions in vitiligo model mice, reduces the degree of pigmentation loss, and decreases infiltration of CD8+ T cells (9). Mechanistic investigations revealed that LBP modulates the 70 kDa heat shock protein (Hsp70)-C-X-C motif chemokine ligand 9/10 (CXCL9/10) signaling axis, suppressing the expression of CXCL9, CXCL10, C-X-C motif chemokine receptor 3 (CXCR3), and HSP70. Moreover, LBP reduces levels of pro-inflammatory cytokines including IL-8, IL-6, TNF-α, interferon-gamma (IFN-γ), and IL-1β, and inhibits STAT3 phosphorylation (9). LBP has a significant impact on splenic immunity in septic animals, particularly affecting T cell responses. The study demonstrated that LBP (*i.g.*, 200, 400, and 800 mg/kg) could improve the proportions of T cell subsets in septic rats, increasing the percentages of CD3+, CD4+, and CD8+ T cells. Notably, LBP produced the most pronounced restoration of T cell populations (10). Compared with the control group, LBP treatment led to a significant increase in T cell numbers, indicating that LBP enhances cellular immunity in septic rats (10). Additionally, LBP improved splenic architecture and reduced spleen damage caused by sepsis, demonstrating strong immunoregulatory and tissue repair potential. These findings suggest that LBP exerts its effects by modulating T cell composition and enhancing splenic immune function (10). In experimental autoimmune encephalomyelitis (EAE) model mice, LBP was shown to improve immune function by modulating CD4+ T cell activation (11). Experimental data indicated that LBP (*i.g.*, 1, 5 and 10 mg/kg *in vivo*; 200 μg/mL *in vitro*) suppresses the differentiation of pathogenic CD4+ T cells, particularly T-helper 1 (Th1) and Th17 subsets, while promoting the polarization of regulatory T cells (Tregs) (11). Furthermore, LBP attenuates inflammatory responses by inhibiting key signaling pathways involved in T cell activation and differentiation, such as the AP-1 and nuclear factor-kappa B (NF-κB) pathways. These results indicate that LBP not only suppresses immunopathological responses but also mitigates pro-inflammatory cytokine production by modulating the balance of CD4+ T cell subsets (11).

#### 2.1.3 Promotion of B cell activation

B cells, also known as B lymphocytes, are a type of white blood cell that play a crucial role in adaptive immune responses. They are responsible for producing high-affinity antibodies, generating immunological memory, functioning as antigen-presenting cells, and secreting cytokines (12). LBP has been shown to modulate immune responses and gut microbiota composition in cyclophosphamide (CTX)-induced immunosuppressed model mice. The results demonstrated that LBP (*i.g.*, 50, 100 and 200 mg/kg), obtained by water extraction and alcohol precipitation, increased immune organ indices, ameliorated organ damage, and promoted the production of immune-related cytokines such as IL-2, IL-6, TNF-α, and IFN-γ (13). Additionally, LBP (*i.g.*, 100 mg/kg) treatment enhanced the production of short-chain fatty acids (SCFAs) and altered the composition of gut microbiota, increasing the relative abundance of *Bacteroidaceae*, *Lactobacillaceae*, *Prevotellaceae*, and *Verrucomicrobiaceae*—microbial families closely associated with immune regulation. LBP may exert its immunoenhancing effects by modulating the interaction between gut microbiota and host immune responses (13). LBP also attenuates cytotoxicity of mice epithelial cells caused by Pseudomonas aeruginosa, inhibiting cell death and oxidative stress induced by its secreted toxin, pyocyanin (PCN). Specifically, LBP (*i.g.*, 3 mg/kg) reduces intracellular reactive oxygen species (ROS) levels, inhibits PCN-induced apoptosis, downregulates the expression of pro-apoptotic protein caspase-3, and enhances the activation of the anti-apoptotic protein Bcl-2 (14). Moreover, LBP suppresses the secretion of PCN-induced inflammatory cytokines, including IL-1β, TNF, IL-6, and IL-8, thereby mitigating tissue damage in the lungs and spleen. These findings provide scientific support for the potential application of LBP in treating respiratory infections (14).

#### 2.1.4 Enhancement of natural killer cell activity

Natural killer (NK) cells are a critical component of the innate immune system and belong to the lymphocyte lineage. Unlike other lymphocytes, they could rapidly recognize and eliminate abnormal cells, such as tumor cells and virus-infected cells, without prior antigen sensitization (15). The study on Hyland Brown laying hens immunosuppressed induced CTX has shown that LBP (*i.g.*, 10, 5, and 2.5 mg/kg) upregulates the expression of immune-related genes, improves body weight and spleen index, and enhances the proliferative capacity of peripheral blood lymphocytes. LBP enhances immune function by modulating key immune-related signaling pathways, including chemokine signaling, C-type lectin receptor signaling, and B cell receptor signaling pathways (16). Metabolomic analysis revealed that LBP (*i.g.*, 5 mg/kg) influences splenic metabolism in immunosuppressed hens, particularly in the glycerophospholipid metabolic pathway. This suggests that LBP may restore immune function by regulating critical metabolic processes involving lipids, amino acids, and energy metabolism (16). Furthermore, LBP3, a purified fraction of LBP from 40 kDa to 350 kDa, markedly alleviates doxorubicin (Dox)-induced BALB/c mice immunosuppression by restoring peripheral blood lymphocyte counts and improving the cell cycle progression of bone marrow cells at oral dose of 250 mg/kg. LBP3 also restores NK cell cytotoxicity and enhances their anti-tumor activity (17). In the H22 tumor-bearing model mice, the combination of LBP3 (*i.g.*, 250 mg/kg) and Dox (*intraperitoneal (i.p.)*, 5 mg/kg) enhances antitumor efficacy of Dox and markedly suppresses tumor growth. Specifically, the tumor inhibition rate reached 79.06% in the combination group, higher than the 51.99% observed in the Dox-alone group. These findings indicate that LBP3 not only mitigates Dox-induced immunotoxicity but also enhances its antitumor efficacy, suggesting its potential as an adjuvant in chemotherapy (17).

#### 2.1.5 Promotion of dendritic cell activity

Dendritic cells (DCs) are the most potent professional antigen-presenting cells (APCs) and serve as a crucial bridge between innate and adaptive immunity. Named for their characteristic dendritic projections, DCs possess strong capacities for antigen uptake, processing, and presentation, and play a central regulatory role in initiating T cell-mediated immune responses (18). The study on the metabolic effects of LBP3 on DCs has shown that LBP3 is non-toxic to mouse bone marrow derived dendritic cell line DC2.4 cells at dose of 50, 100 and 200 μg/mL and promotes the secretion of cytokines such as IL-6, IL-12, and TNF-α (19). Metabolomic analysis revealed that LBP3 treatment altered 20 differential metabolites in DCs, involving 28 metabolic pathways, primarily including aspartate metabolism, pyrimidine metabolism, and fatty acid metabolism. These findings suggest that LBP may promote the maturation and immune functionality of DCs by modulating metabolic pathways, offering new insights into its immunostimulatory mechanisms (19). Investigations into the molecular mechanisms of LBP-induced DC maturation in mice revealed that LBP (50, 100 and 200 μg/mL) upregulates the surface expression of major histocompatibility complex class II (MHC-II), CD80, and CD86, and enhances the secretion of IL-6 and IL-4. LBP promotes DC maturation by upregulating Toll-like receptor 4 (TLR4) and activating the extracellular regulated kinase 1/2 (ERK1/2) signaling pathway, thereby enhancing the expression of the transcription factor B lymphocyte maturation-induced protein-1 (Blimp1) (20). The TLR4 inhibitor TAK242 and ERK1/2 inhibitors block this signaling pathway and reduce Blimp1 expression, while siRNA-mediated Blimp1 knockdown also decreases IL-6 levels. These results suggest that LBP regulates DC maturation through the TLR4- ERK1/2-Blimp1 signaling axis, providing a molecular basis for its immunomodulatory activity (20). Interestingly, neutral *Lycium barbarum* polysaccharides (NLBP) and acidic *Lycium barbarum* polysaccharides (ALBP) extracted from *Lycium barbarum* exhibit distinct physicochemical properties and immunoregulatory effects. NLBP has a higher MW, greater mobility, and lower thermal stability compared to ALBP, and demonstrates superior immunostimulatory activity by promoting DC maturation and inducing a decrease in CD206 and increase the level of CD80. *In vivo* experiments further confirmed that NLBP (*i.p.*, 2 mg/mL) inhibits melanoma growth by enhancing macrophage phagocytic activity, achieving a tumor inhibition rate of up to 66.7% (21). This study highlights a potential correlation between the structural characteristics of LBP and its biological activities.

### 2.2 Regulation of immune responses

#### 2.2.1 Effects of LBP on cytokine secretion

Cytokines are small protein molecules secreted by immune cells (e.g., T cells, macrophages, dendritic cells) and non-immune cells (e.g., endothelial cells, fibroblasts), functioning as key signaling molecules in the immune system (22). A study has shown that LBP solution (1% (w/v)) activates the nuclear factor E2-related factor 2 (Nrf2) signaling pathway in a DSS-induced chronic ulcerative colitis (UC) model mice, promotes the expression of tight junction proteins (ZO-1 and Occludin), and suppresses Claudin-2, thereby restoring intestinal barrier integrity. Additionally, LBP modulates gut microbiota by increasing the abundance of beneficial bacteria (such as *Lactobacillus* and *Akkermansia*) and short-chain fatty acids (SCFAs), while reducing pathogenic bacteria (such as *Sutterella* and *Mucispirillum*), demonstrating a pronounced prebiotic effect (23). Researchers developed LBP-derived nanoparticles (PLBP) for treating retinal ischemia–reperfusion (IR) injury. Results showed that PLBP (intravitreal injection, 10 mg/mL) alleviated ferroptosis and oxidative damage in retinal ganglion cells in IR model mice by scavenging ROS and activating the Nrf2 antioxidant pathway. It also inhibited microglial activation and cytokine release by suppressing the NF-κB signaling pathway (24). Another study evaluated the effect of LBP on promoting corneal re-epithelialization after alkali burn injury. Mice pretreated with 2 mg/mL LBP eye drops for seven days prior to injury exhibited accelerated corneal epithelial healing, reduced corneal opacity, decreased expression of inflammatory cytokines (MMP12, IL-1β, PDGF-BB) and aquaporin (AQP5), without inducing apoptosis. These findings highlight the anti-inflammatory and corneal-protective properties of LBP, suggesting its potential as a natural therapeutic and preventive agent for ocular chemical injuries (25). The roles of LBP in prebiotic activity, immune stimulation, and gut microbiota modulation are receiving increasing research attention. *In vitro*, LBP (2.5%, 5%, 10%, 15% (w/v)) promotes the growth of *Lactobacillus acidophilus* and *Bifidobacterium longum*. *In vivo*, LBP solution (0.1 mL/10 g) increased the abundance of probiotic genera in mice, such as *Akkermansia* and *Lactobacillus*, and elevated levels of transforming growth factor beta (TGF-β), IL-6, and intestinal secretory immunoglobulin A (SIgA), thereby enhancing innate immunity (26). These findings support the potential of LBP as a natural prebiotic with dual functions in gut microbiota modulation and immune enhancement. The herbicide 2,4-D triggers BV2 microglial cells neuroinflammation by inducing excessive mitochondrial ROS production, activating the nod-like receptor protein 3 (NLRP3) inflammasome, impairing autophagy, and promoting the release of inflammatory cytokines such as TNF-α, IL-1β, IL-6, and IL-18 (27). In contrast, LBP (300 μg/mL) reduces ROS production, downregulates the expression of NLRP3 inflammasome components and inflammatory cytokines, and restores levels of light chain 3 II (LC3II) and Beclin-1 in BV2 microglial cells, thereby enhancing autophagic activity and exerting neuroprotective effects (27). A double-blind, randomized, placebo-controlled clinical trial investigated the antidepressant effect of LBP in adolescents and its underlying immunological mechanisms. The results indicated that daily intake of 300 mg LBP for six weeks reduced depressive symptoms in adolescents and decreased levels of the pro-inflammatory cytokine IL-17A in peripheral blood (28). Network analysis further revealed that LBP reduced systemic inter-cytokine correlations, suggesting that its antidepressant effect may be mediated through modulation of inflammatory responses (28).

#### 2.2.2 Regulation of immune tolerance and immune memory by LBP

Immune tolerance refers to the state in which the immune system does not mount a response to specific antigens, such as self-antigens or dietary antigens, and is a key mechanism for maintaining physiological homeostasis (29). Immune memory is a hallmark of the adaptive immune system, enabling the host to mount a rapid and effective protective response upon re-exposure to the same pathogen (30). A study has reported that the unique structure of Pickering emulsions allows LBP to combine with aluminum-based adjuvants, enhancing antigen loading capacity and stability. In the immunization experiment on ICR mice, LBP-based Pickering emulsions (LBPPE) (*s.c.*, 200 μg per mouse) markedly promoted the recruitment of APCs and activation of dendritic cells, enhanced the production of antigen-specific immunoglobulin G (IgG), IgG1, and IgG2a antibodies, and elicited robust cellular immune responses, including the induction of memory T cell responses (31). LBPPE also exhibited stronger immunostimulatory effects than conventional aluminum adjuvants, including more sustained antigen release and more pronounced cell-mediated immune responses, particularly in enhancing vaccine efficacy and immunological memory (31). Furthermore, administration of LBP (*i.g.*, 5 and 10 mg/kg) improved salivary gland function and reduced lymphocytic infiltration in primary Sjogren’s syndrome (pSS) model mice. It also modulated T cell subset balance by suppressing effector T cells (e.g., T follicular helper and Th17 cells) and promoting regulatory T cells, thereby slowing the progression of pSS (32). The immunoregulatory effect of LBP was further demonstrated by its ability to reduce serum levels of autoantibodies Sjogren’s-syndrome-related antigen A (SSA/Ro) and Sjogren’s-syndrome-related antigen B (SSB/La), thus alleviating autoimmune responses (32). These findings suggest that LBP may serve as a promising therapeutic candidate for pSS, particularly in the context of immune modulation and anti-inflammatory intervention.

#### 2.2.3 Regulation of complement activity

The complement system, composed of over 30 plasma proteins, is a sophisticated cascade reaction mechanism that plays a critical role in bridging innate and adaptive immunity (33). A study has shown that LBP exert protective effects against PM2.5-induced skin damage. LBP (2.5 mg/mL) effectively alleviated PM2.5-induced cytotoxicity and apoptosis in HaCaT cells by inhibiting oxidative stress, endoplasmic reticulum (ER) stress, and autophagy pathways (34). Specifically, LBP treatment reduced ROS production and malondialdehyde (MDA) levels in HaCaT cells, while enhancing superoxide dismutase (SOD) activity following PM2.5 exposure. Moreover, LBP mitigated ER stress-induced cellular damage by downregulating the expression of ER stress markers glucose-regulated protein 78 (GRP78) and C/EBP homologous protein (CHOP). In addition, LBP inhibited PM2.5-induced autophagy, thereby further reducing apoptosis (34). Interestingly, LBP also exhibited significant effects on immune parameters, apoptosis, and growth performance in Oreochromis niloticus (Nile tilapia). Dietary supplementation with LBP (500, 1000, 1500 and 2000 mg/kg) increased complement component 3 (C3) activity, upregulated IL-1β gene expression in the spleen, reduced apoptosis in splenic tissues, and improved specific growth rate (SGR), relative length gain (LG), and relative weight gain (WG) in fish (35). However, the study noted that LBP had minimal effects on serum alkaline phosphatase (AKP), MDA levels, and SOD activity in Nile tilapia. Immune-related gene analysis revealed that IL-1β expression in the spleen was upregulated in all LBP treatment groups, with the most significant increase observed at a supplementation level of 500 mg/kg. These findings suggest that LBP could enhance non-specific immune responses, reduce cellular apoptosis, and promote growth and development in Oreochromis niloticus (35) (**Table 1**).

**Table 1:** Effects and mechanisms of LBP on the immune system.

Animals or cells	Disease model	Dosage of administration	Administration time	Mechanisms	References
C57BL/6 male mice	Colitis	*i.g.*, 200 mg/kg	9 days	Reduced M1 polarization by inhibiting STAT1 phosphorylation and enhanced M2 polarization by promoting STAT6 phosphorylation	(4)
RAW264.7 macrophages	LPS-induced inflammation	50 μg/mL	24 h	Inhibited LPS-induced M1 macrophage differentiation and promoted M2 polarization, concurrently restoring the expression of M2-associated anti-inflammatory cytokines IL-4 and IL-10	(5)
RAW264.7 macrophages	LPS-induced inflammation	400 and 800 μg/mL	48 h	Reduced the secretion of pro-inflammatory cytokines TNF-α, IL-1β, IL-6 and NO	(7)
C57BL/6 female mice	Vitiligo model mice	*i.g.*, 0.3 and 0.6 g/kg	8 weeks	Modulated the Hsp70- CXCL9/10 signaling axis, suppressed the expression of CXCL9, CXCL10, CXCR3, and HSP70	(9)
Sprague Dawley male rats	Septicemia	*i.g.*, 200, 400, and 800 mg/kg	2 weeks	Improved the proportions of T cell subsets in septic rats, and increased the percentages of CD3+, CD4+, and CD8+ T cells	(10)
Female C57BL/6J mice	Experimental autoimmune encephalomyelitis	*i.g.*, 1, 5 and 10 mg/kg	20 days	Suppressed the differentiation of pathogenic CD4+ T cells, particularly Th1 and Th17 subsets, while promoting the polarization of regulatory T cells (Tregs)	(11)
Male BALB/c mice	CTX-induced immunosuppressed model	*i.g.*, 50, 100 and 200 mg/kg	19 days	Increased immune organ indices, ameliorated organ damage, promoted the production of immune-related cytokines such as IL-2, IL-6, TNF-α, and IFN-γ, and enhanced the production of short-chain fatty acids and alter the composition of gut microbiota	(13)
Bronchial epithelial cells	Cytotoxicity caused by Pseudomonas aeruginosa	*i.g.*, 3 mg/kg	2 h	Reduced intracellular ROS levels, inhibited PCN-induced apoptosis, downregulated the expression of pro-apoptotic protein caspase-3, and enhanced the activation of the anti-apoptotic protein Bcl-2	(14)
Hyland Brown laying hens	CTX-induced immunosuppressed model	*i.g.*, 10, 5, and 2.5 mg/kg	28 days	Upregulated the expression of immune-related genes, improved body weight and spleen index, and enhanced the proliferative capacity of peripheral blood lymphocytes	(16)
BALB/c mice	H22 tumor-bearing model	*i.g.*, 250 mg/kg	10 days	Restored peripheral blood lymphocyte counts and improved the cell cycle progression	(17)
C57BL/6J male mice	DSS-induced chronic ulcerative colitis	1% LBP solution (w/v)	1 weeks	Activated the Nrf2 signaling pathway in a DSS-induced chronic ulcerative colitis mouse model, promoted the expression of tight junction proteins, and suppressed Claudin-2, thereby restoring intestinal barrier integrity	(23)
Male C57BL/6 mice	Ischemia reperfusion	Intravitreal injection, 10 mg/mL	Once	Scavenged ROS and activated the Nrf2 antioxidant pathway	(24)
Adolescents	Depression	300 mg/day	6 weeks	Reduced depressive symptoms in adolescents and decreased levels of the pro-inflammatory cytokine IL-17A in peripheral blood	(28)
Female NOD mice	Primary Sjogren’s syndrome	*i.g.*, 5 and 10 mg/kg	8 weeks	Improved salivary gland function and reduced lymphocytic infiltration	(32)
Female ICR mice	Estrogen deprivation model	*i.g.*, 100 mg/kg	24 weeks	Suppressed neuroinflammatory responses by downregulating the TLR4/NF-κB signaling pathway	(38)
Male ICR mice	Lead-induced renal injury	*i.g.*, 400 mg/kg	5 weeks	Inhibited TLR4 activation, thereby suppressing the PI3K/Akt/mtor signaling pathway	(39)
C57BL/6 male mice	Colorectal cancer	*s.c.*, 200 μg	35 days	Enhanced dendritic cell uptake and maturation via the synergistic activation of TLR4 and MGL signaling pathways, thereby boosting CD8+ T cell cytotoxicity.	(40)
SD male rats	Rheumatoid arthritis	*i.g*., 200 and 400 mg/kg	28 days	Inhibited TLR4/NF-κB-mediated inflammatory signaling	(41)
BV2 microglia	LPS induced amoeboid-like activation	0.6, 0.9 and 1.2 g/L	24 h	Inhibited LPS-induced activation of BV2 microglial cells and promoted their polarization from the pro-inflammatory M1 phenotype to the anti-inflammatory M2 phenotype via suppression of the TLR4/NF-κB signaling pathway	(42)
Bovine mammary epithelial cells	LPS-induced inflammation	100 and 300 μg/mL	24 h	Activated the PPARγ receptor and inhibited the activation of MAPK and NF-κB signaling pathways	(46)
C57BL/6 female mice	Ulcerative colitis	*i.g.*, 100 mg/kg	10 days	Promoted the expression of tight junction proteins (tjs), activated the Nrf2/HO-1 antioxidant pathway, and inhibited the NF-κB signaling pathway	(48)
BALB/c mice	Allergic rhinitis	*i.g.*, 25, 50 and 100 mg/kg	21 days	Inhibited the expression of TLR4/NF-κB pathway-related proteins, restored Th1/Th2 cytokine balance, and reduced IgE production	(49)
SD female rats	Intracranial aneurysms	i.g., 5, 10 and 20 mg/kg	30 days	Suppressed the TLR4/NF-κB pathway and reduced inflammatory responses	(50)
Female BALB/c mice	Immunosuppressive model	*i.g*., 100 mg/kg	10 days	Inhibited activation of the MLCK signaling pathway and reduced the expression of MLCK and phosphorylated MLC proteins	(51)
Human colorectal cancer Caco-2 cells	Intestinal barrier dysfunction	400 μg/mL	24 h	Restored the expression of tight junction proteins, and inhibited MLCK and MLC phosphorylation	(52)
Male C57BL/6 mice	Hepatic encephalopathy	*i.g*., 5 mg/kg	7 days	Inhibited the MAPK pathway and modulated inflammatory signaling between the liver and brain	(54)

## 3 Molecular mechanisms of LBP-mediated immune regulation

### 3.1 TLR pathway

Toll-like receptors (TLRs), a key family of pattern recognition receptors (PRRs) in the innate immune system, play a central role in initiating immune responses, regulating inflammation, and bridging innate and adaptive immunity by recognizing pathogen-associated molecular patterns (PAMPs) and damage-associated molecular patterns (DAMPs) (36). The study has shown that LBP (50, 100 and 200 μg/mL) promotes the maturation of DCs in mice via the TLR4–ERK1/2–Blimp1 signaling pathway. LBP upregulates the expression of surface markers MHCII, CD80, and CD86 on DCs, and enhances the secretion of cytokines such as IL-6 and IL-4 (20). The immunomodulatory effects of LBP depend on the activation of TLR4, which subsequently triggers the ERK1/2 and Blimp1 signaling molecules. The use of the TLR4 inhibitor TAK-242 and the ERK1/2 inhibitor PD98059 attenuated LBP-induced DC maturation. Moreover, Blimp1 knockdown reduced IL-6 secretion, suggesting a pivotal role for Blimp1 in LBP-mediated DC maturation (20). The immunomodulatory effects of LBP are influenced by its MW and gastrointestinal digestion. It has been found that the MW of LBP affects its binding affinity to the TLR4/myeloid differentiation protein-2 (MD-2) receptor complex and its ability to induce cytokine production. LBP of medium MW (100 kDa-300 kDa) exhibits higher binding affinity and cytokine-inducing activity, while LBP with a MW below 10 kDa shows markedly reduced immunological activity (37). Simulated gastrointestinal digestion degrades LBP into smaller fragments, which, despite reduced binding affinity, retain the ability to stimulate cytokine production (37). Estrogen deprivation (OVX) leads to significant learning and memory impairments in ICR mice, which are markedly ameliorated by LBP treatment. Experimental results show that LBP (*i.g.*, 100 mg/kg) alleviates neuronal damage in the CA1 and CA3 regions of the hippocampus in mice. LBP suppresses neuroinflammatory responses by downregulating the TLR4/NF-κB signaling pathway, thereby reducing the expression of pro-inflammatory cytokines such as IL-6, IL-1β, and TNF-α (38). LBP exhibits protective effects against lead-induced renal injury in mice, mitigating weight loss, renal dysfunction, and tissue damage caused by lead exposure. Mechanistically, LBP (*i.g.*, 400 mg/kg) inhibits TLR4 activation, thereby suppressing the phosphatidylinositol 3-kinase (PI3K)/protein kinase B (Akt)/mammalian target of the rapamycin (mTOR) signaling pathway, enhancing autophagy, and reducing apoptosis in renal cells, ultimately alleviating lead-induced kidney injury (39). Additionally, LBP ameliorates hematological abnormalities induced by lead exposure and restores normal red blood cell and platelet function of mice (39).

Researchers have developed a CD155 gene-loaded liposomal nanovaccine modified with LBP (LBP-CD155L NVs), and the immune checkpoint PD-1/PD-L1 were also explored to enhance immunotherapy for colorectal cancer (CRC) (40). *In vitro* experiments demonstrated that LBP-CD155L NVs (200, 150, 100, 50 and 25 μg/mL) could target and upregulate the expression of activation and maturation markers on the surface of DCs, such as CD11c+, CD86+ and CD80+, thereby promoting DCs maturation. Silencing of galactose type lectins (MGL) attenuated LBP-induced activation of the MAPK pathway, substantiating that LBP induces DCs maturation through its interaction with MGL. Furthermore, LBP-CD155L NVs were found to induce the mRNA expression of Batf3 and Irf4 in DCs, an effect that was diminished upon MGL silencing. These findings indicated that LBP orchestrates DC maturation through the synergistic engagement of MGL and TLR4 pathways. Notably, LBP-CD155L NVs enhanced the frequency of CD8+ T cells induced by bone marrow-derived dendritic cells (BMDCs), and elevated the secretion of IFN-γ and TNF-α by CD8+ T cells. Concurrently, LBP potentiated the cytotoxicity of DC-induced tumor-reactive effector T cells. Through this mechanism, LBP-CD155L NVs improve the efficacy of immune checkpoint blockade (ICB) therapy, promote T cell tumor infiltration, and suppress the immunosuppressive tumor microenvironment (40). *In vivo* experiments demonstrated that LBP-CD155L NVs (*subcutaneous (s.c.)*, 200 μg) exert both prophylactic and therapeutic effects against transplanted HT-29 tumors in C57BL/6 mice. LBP-CD155L NVs increased the proportion of mature DCs within peripheral immune organs and elevated the levels of TNF-α and IFN-γ secreted by infiltrating CD8+ T cells, while concomitantly reducing the frequency of PD-1 expression. Notably, LBP-CD155L NVs induced a significant increase in the CD86 level and a concomitant decrease in CD206 level within the tumor micro environment (TME). Compared to treatment with αPD-1 antibody alone, the combination of LBP-CD155L NVs and αPD-1 markedly enhances antitumor immune responses and therapeutic outcomes. Combination immunotherapy enhances antitumor immune responses by promoting DCs maturation and the differentiation of the cDC1 subset, as well as by reprogramming macrophage polarization, reducing the myeloid-derived suppressor cells (MDSCs), and augmenting immune cell infiltration within the TME, thereby ameliorating the immunosuppressive milieu associated with immunotherapy (40). In a study investigating the effects and mechanisms of LBP in rheumatoid arthritis (RA) model rats, LBP (*i.g.*, 200 and 400 mg/kg) treatment reduced paw swelling and arthritis scores, increased mechanical pain threshold and thermal withdrawal latency, alleviated histopathological changes in synovial tissues, and lowered serum levels of TNF-α, IL-1β, and IL-6. Furthermore, mRNA expression of TNF-α, IL-1β, and IL-6, as well as protein expression of TLR4, MyD88, and p-NF-κB in synovial tissues, were markedly reduced. These findings suggest that LBP ameliorates RA symptoms by inhibiting TLR4/NF-κB-mediated inflammatory signaling (41). A study indicated that LPS induces amoeboid-like activation of BV2 microglia, leading to increased NO release and elevated levels of Iba-1, TLR4, NF-κB, inducible nitric oxide synthase (iNOS), TNF-α, IL-1β, and IL-6, while reducing the levels of Arg1 and IL-10. LBP (0.6, 0.9 and 1.2 g/L) reverses the expression of Iba-1, TLR4, NF-κB, iNOS, TNF-α, IL-1β, and IL-6 in a dose-dependent manner in BV2 microglial cells, while increasing Arg1 and IL-10 levels in a dose-dependent manner. These findings suggest that LBP may inhibit LPS-induced activation of BV2 microglial cells and promote their polarization from the pro-inflammatory M1 phenotype to the anti-inflammatory M2 phenotype via suppression of the TLR4/NF-κB signaling pathway (42). It has been reported that LBP (*i.g.*, 100 mg/kg) effectively modulates Th1/Th2 cytokine balance and alleviates eosinophilic inflammation in the nasal mucosa of rats with allergic rhinitis, possibly through inhibition of the TLR9/AP-1 signaling pathway (43). Additionally, LBP (200, 100 and 50 mg/L) regulates the activation of RAW264.7 macrophages via the TLR2/TLR4/MGL pathways, thereby participating in the immune response (44).

### 3.2 NF-κB pathway

**Figure 2:**
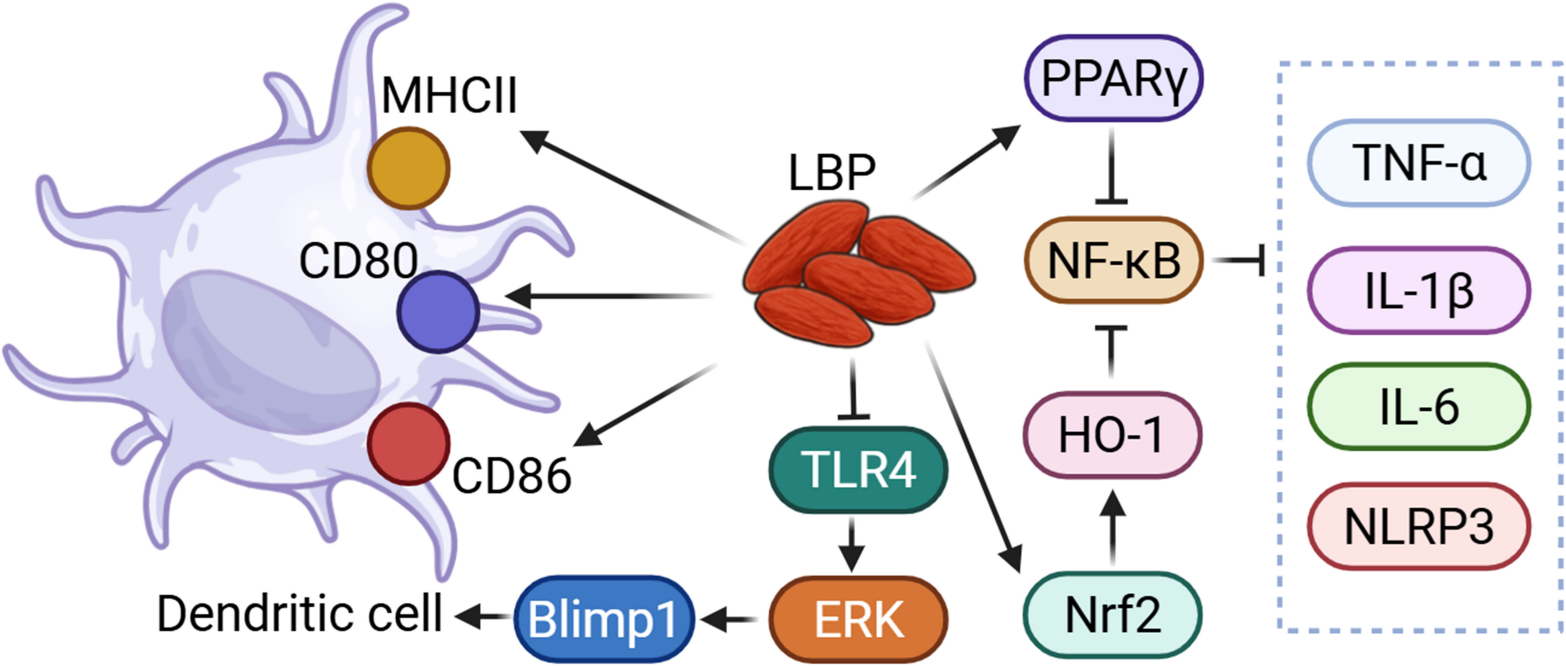
LBP could regulate immune function through TLR and NF-κB related pathway. LBP could modulate dendritic cells via the TLR4/ERK/Blimp1 signaling pathway. Additionally, LBP could suppress NF-κB activity through the activation of PPARγ and the Nrf2/HO-1 pathway, thereby reducing the levels of pro-inflammatory cytokines and inflammasomes.

NF-κB is one of the most pivotal transcription factors in immune responses, playing a key role in regulating inflammation, cell survival, and immune cell development, and is critically involved in infections, tumors, and autoimmune diseases (45). The study has shown that LBP (100 and 300 μg/mL) alleviates LPS-induced inflammatory responses of bovine mammary epithelial cells (bMECs) by activating the peroxisome proliferator-activated receptor gamma (PPARγ) receptor and inhibiting the activation of mitogen-activated protein kinase (MAPK) and NF-κB signaling pathways (46). Additionally, LBP treatment enhances PPARγ protein expression and reduces cellular inflammation by downregulating pro-inflammatory cytokines such as TNF-α, IL-1β, and IL-6, as well as suppressing cyclooxygenase (COX)-2 and NLRP3 protein levels. LBP also counteracts the inhibitory effects of LPS on the proliferation and migration of bMECs, thereby promoting cellular repair (46). An interesting study reported that aspirin increases the secretion of pro-inflammatory cytokines TNF-α and IL-6, and activates the NF-κB and ERK1/2 signaling pathways in rat normal gastric mucosa RGM-1 cells. Pretreatment cells with LBP (100-500 μg/mL) reduced TNF-α and IL-6 secretion and inhibits NF-κB activation. LBP also reduced apoptosis in gastric mucosal cells, lower Bax protein expression, and mitigate apoptosis by inhibiting c-Jun N-terminal kinase (JNK) activation. Combined treatment with C-phycocyanin and LBP exhibits synergistic anti-inflammatory and anti-apoptotic effects, with potential gastroprotective properties (47). Arabinogalactan polysaccharide (LBP-m) with a MW of 172 kDa isolated and characterized from *Lycium barbarum* could alleviate DSS-induced UC mice. Mechanistically, LBP-m (*i.g.*, 100 mg/kg) promotes the expression of tight junction proteins (TJs), activates the Nrf2/Heme oxygenase-1 (HO-1) antioxidant pathway, and inhibits the NF-κB signaling pathway to reduce mucosal damage and inflammation in UC mice (48). LBP-m also improves gut health by modulating the intestinal microbiota and promoting the production of short-chain fatty acids (SCFAs). Moreover, LBP-m exhibits high thermal stability and a random coil structure, making it suitable for applications in food and pharmaceutical industries (48). LBP regulates the NF-κB signaling pathway to modulate Th1/Th2 cytokine levels in mice with allergic rhinitis. Experimental results showed that LBP (*i.g.*, 25, 50 and 100 mg/kg) reduces nasal symptom scores of allergic rhinitis model mice, alleviates mucosal inflammation and glandular hyperplasia, decreases serum IL-4 and IgE levels, and lowers the expression of TLR4 and phosphorylated NF-κB p65 in nasal tissues, while increasing serum IFN-γ levels in a dose-dependent manner. These findings suggest that LBP alleviates nasal mucosal inflammation in allergic rhinitis by inhibiting the expression of TLR4/NF-κB pathway-related proteins, restoring Th1/Th2 cytokine balance, and reducing IgE production (49). In a study on intracranial aneurysms (IA) model rats, LBP (*i.g.*, 5, 10 and 20 mg/kg) led to the flattening of bulges in the Circle of Willis, reduction in vessel wall thickness and aneurysm volume, and attenuation of pathological changes such as endothelial cell vacuolization and inflammatory cell infiltration. Serum levels of IL-6, TNF-α, vascular endothelial growth factor (VEGF), and endothelin (ET), as well as NF-κB and TLR4 protein expression in aneurysmal vascular tissues, were reduced. These results suggest that LBP may alleviate endothelial damage in IA model rats by suppressing the TLR4/NF-κB pathway and reducing inflammatory responses (50) (**Figure 2**).

### 3.3 Others

LBP also modulates immune function through additional molecular mechanisms. It improves intestinal barrier function and immune responses by inhibiting the myosin light chain kinase (MLCK) signaling pathway. A study has demonstrated that LBP (*i.g.*, 100 mg/kg) enhances digestive and absorptive functions in immunosuppressive model mice, increases D-xylose excretion, and enhances the phagocytic activity of peritoneal macrophages. LBP also alleviates intestinal barrier dysfunction in immunosuppressive model mice, reduces intestinal permeability, and enhances immune function by modulating levels of immunoglobulins such as IgA, IgG, and IgM. Inflammatory cytokine levels, including IL-6, IL-12, and IFN-γ, are also improved (51). These findings suggest that LBP inhibits activation of the MLCK signaling pathway, reducing the expression of MLCK and phosphorylated MLC proteins (51). Another related study found that LBP extracted by water extraction and alcohol precipitation with a MW of 3500 kDa could improve TNF-α-induced intestinal barrier dysfunction and inflammation via the MLCK-MLC signaling pathway. *In vitro* study to Caco-2 cells, LBP (400 μg/mL) reduces TNF-α-induced increases in intestinal epithelial permeability and decreases in transepithelial resistance (TER), while restoring the expression of tight junction proteins such as claudin-1, ZO-3, and Occludin. Additionally, LBP suppresses TNF-α-induced secretion of inflammatory cytokines (e.g., IL-6, IL-8, intercellular adhesion molecule 1, and monocyte chemoattractant protein-1), and by inhibiting MLCK and MLC phosphorylation, it attenuates NF-κB activation and further alleviates intestinal barrier damage (52).

LBP has beneficial effects on immunity and liver damage in spotted sea bass exposed to high soybean meal diets (HSBMD). Experimental results showed that HSBMD induces immune suppression and liver damage in spotted sea bass, while LBP (*i.g.*, 1.0, 1.5, 2.0 g/kg) supplementation improves immune function and protects liver health. Specifically, LBP increases the activity of serum lysozyme, IgM, hepatic acid phosphatase, and AKP, while reducing alanine aminotransferase and aspartate aminotransferase levels in serum and liver tissues and improving liver histomorphology (53). Transcriptome analysis revealed that LBP protects the liver by regulating signaling pathways related to glucose and lipid metabolism, such as glycolysis and the insulin signaling pathway (53).

**Figure 3:**
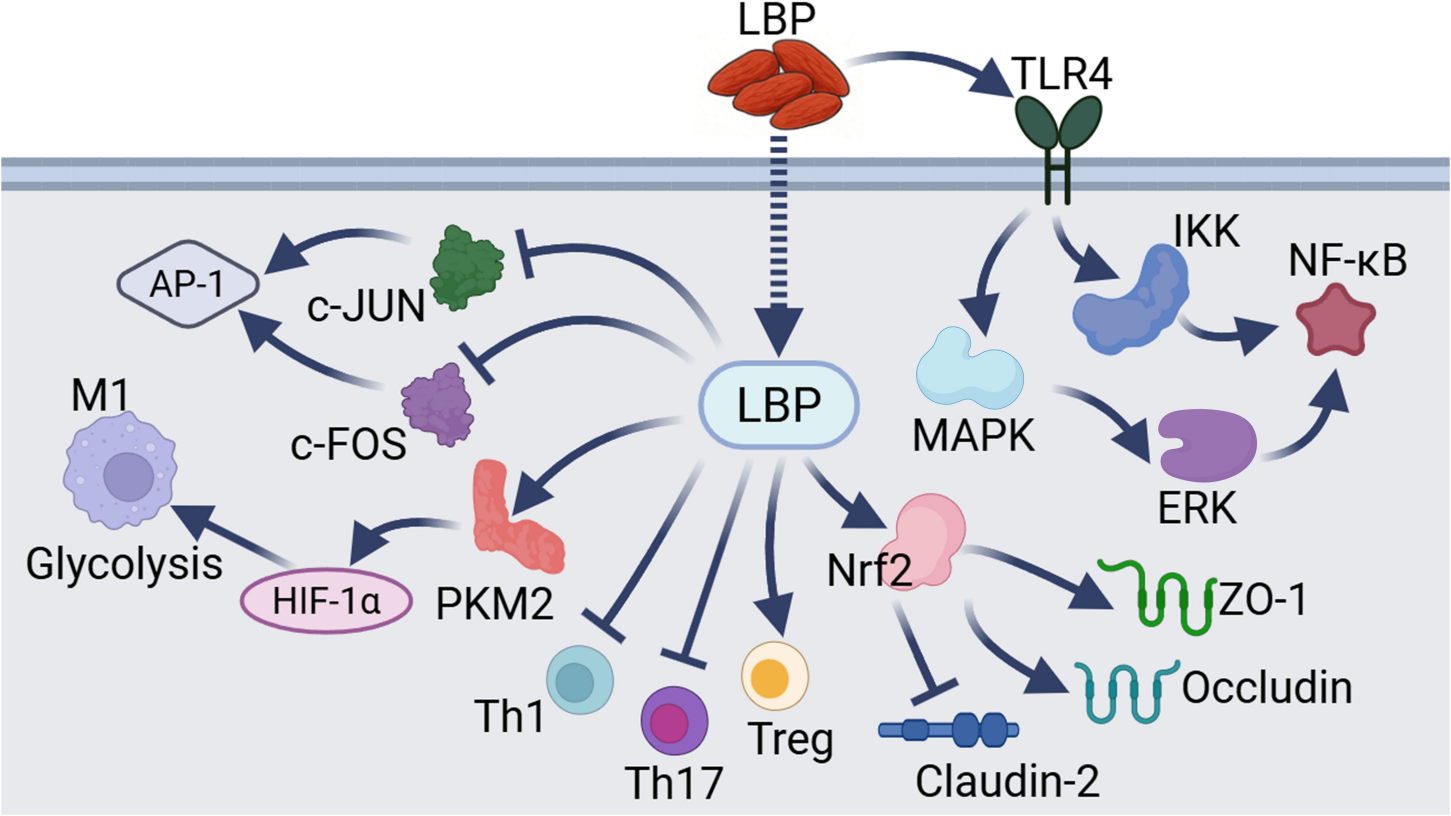
Other mechanisms underlying LBP-mediated immunomodulation. These pathways include the regulation of key targets such as AP-1, glycolysis, Th1, Th17, Treg, junctional proteins, and NF-κB by LBP through distinct mechanisms.

In a study on the protective effects of LBP in hepatic encephalopathy (HE), it was found that LBP alleviates thioacetamide - induced liver injury in HE model mice by inhibiting the MAPK signaling pathway (54). LBP (*i.g.*, 5 mg/kg) ameliorates hepatic tissue damage, reduces oxidative stress and apoptosis, and lowers hepatic levels of pro-inflammatory cytokines TNF-α and IL-6. Further analysis indicates that LBP reduces ammonia and inflammatory cytokine levels in serum and brain, improves behavioral functions in model mice, and alleviates motor dysfunction and depressive symptoms. Moreover, LBP modulates microglial function, mitigating damage induced by ammonia or cytokines. Overall, LBP inhibits the MAPK pathway and modulates inflammatory signaling between the liver and brain, offering a novel approach for the treatment of hepatic encephalopathy (54). (**Table 1**) (**Figure 3**).

## 4 Research on LBP in immune-related diseases

### 4.1 Cancer

Immune function is closely associated with tumor development. The immune system eliminates tumor cells through immune surveillance mechanisms involving NK cells and T cells, while tumors evade immune attacks via mutation accumulation and remodeling of the TME (immune evasion) (55). The latest research showed that mechanisms of immune evasion undermine anti-tumor immunity (56). Locally, these mechanisms act within the TME to directly restrict T cell-mediated cytotoxicity against cancer cells. Tumor cells could suppress the function of anti-tumor T cells by upregulating immune checkpoint molecules. Immune exclusion from the TME results in compromised local immune surveillance, while alterations in the availability of chemokines further constrain T cell activity (57). For example, SOX2 signaling has been implicated in the recruitment of regulatory T cells, and various intrinsic alterations within cancer cells reduce chemotactic signaling, thereby impairing the recruitment of immune cells into the TME and restricting T cell infiltration and motility. On a broader scale, systemic immune evasion mechanisms inhibit the effective priming and establishment of robust T cell responses. The absence of chemokines such as CCL4 and CCL5 leads to the exclusion of dendritic cells from the TME, thereby attenuating the initiation of anti-tumor T cell immunity (58). Collectively, these strategies enable tumors to circumvent immune-mediated eradication, ultimately fostering tumor progression and therapeutic resistance. Therefore, enhancing the immune system offers therapeutic benefits against tumors.

**Figure 4:**
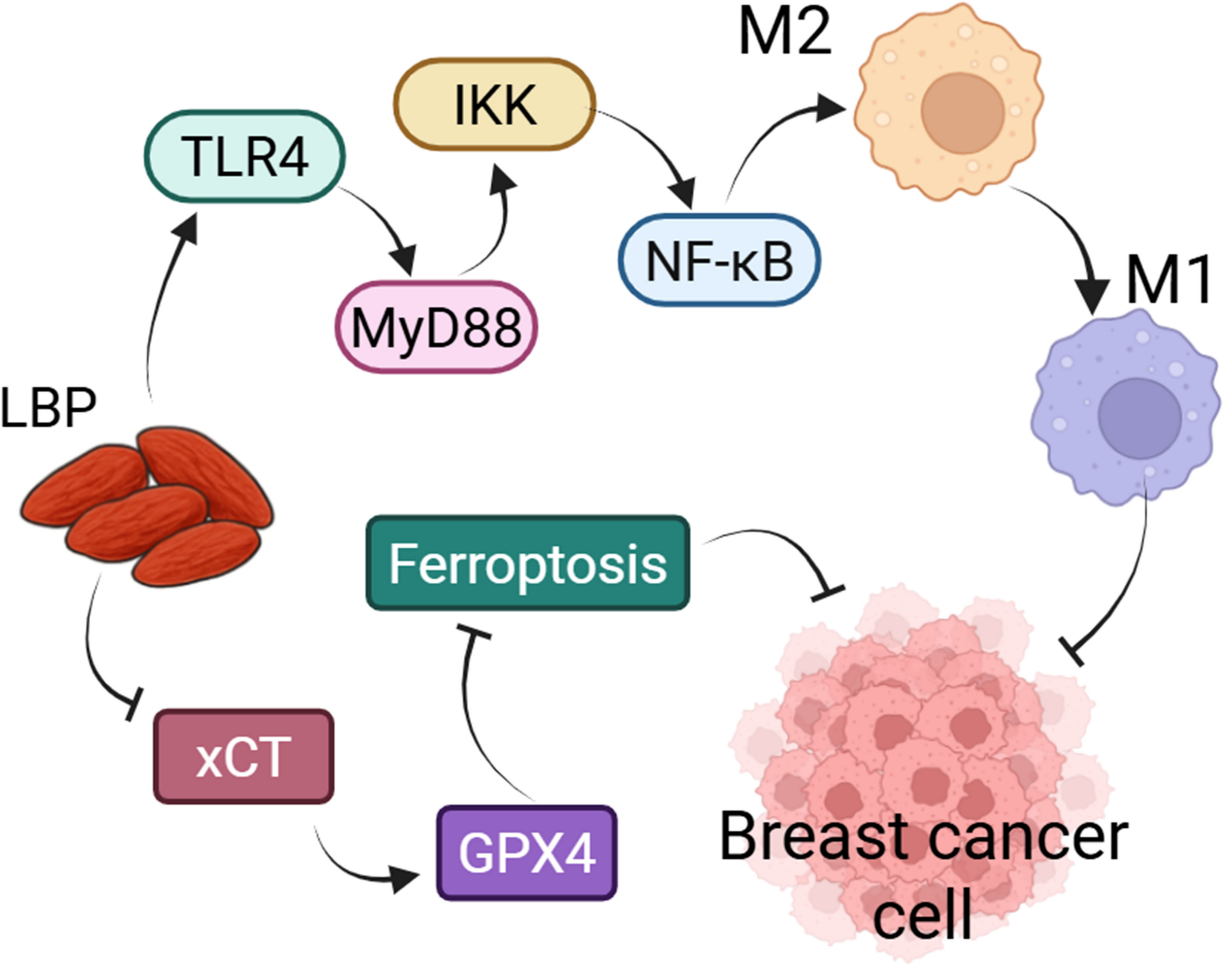
LBP could inhibit the growth of breast cancer cells by regulating macrophage polarization and ferroptosis. LBP could participate in the polarization of macrophages from the M2 to the M1 phenotype via the TLR4/MyD88/IKK/NF-κB signaling pathway, thereby suppressing the proliferation of breast cancer cells. Similarly, LBP inhibits the proliferation of breast cancer cells by modulating ferroptosis through the xCT/GPX4 axis.

LBP shows promising potential in breast cancer treatment, particularly through its modulation of tumor-associated macrophage polarization. A study has shown that LBP with a MW of 50–100 kDa could promote the *Il1β*, *Nos2* and *Tnfα* gene level and inhibit *Arg1*, *Chil3*, *Il4* gene level, enhances phagocytic activity, and thereby inhibits the proliferation of breast cancer cells. In breast cancer model mice, LBP (intratumoral injection, 2 mg/mL) reduced tumor volume and enhanced anti-tumor immune responses. Mechanistically, LBP activates innate immune responses in macrophages via the TLR4-MyD88-IKK-NF-κB signaling pathway and upregulates the expression of immune-related genes such as Trem1, Treml2, and Ly6a (59). These findings suggest that LBP has the potential to enhance anti-tumor immunity by promoting macrophage polarization. Researchers have developed a liposomal nanovaccine LBP-CD155L NVs to enhance immunotherapy efficacy against CRC. The *vitro* and *vivo* studies indicated that LBP-CD155L nanovaccines enhances the uptake and maturation of DCs through the TLR4 and MGL pathways and promotes CD8+ T cell activation. LBP-CD155L NVs reduce immunosuppressive cells in the tumor microenvironment, such as MDSCs and Tregs, while enhancing CD86 level and promoting effector T cell infiltration (40). Moreover, when combined with anti-PD-1 therapy, LBP-CD155L NVs improve immunotherapeutic efficacy, reverse the immunosuppressive tumor microenvironment, and markedly inhibit tumor growth (40). Malignant glioma is the most common primary malignant tumor of the central nervous system, characterized by high invasiveness, frequent recurrence, and poor prognosis (60). A study on the combination of LBP (*i.g.*, 50 mg/kg) with temozolomide (*intravenous (i.v.)*, 150 mg/m^2^) and bevacizumab (*i.v.*, 10 mg/kg) in the treatment of malignant glioma showed that this therapy normalizes tumor morphology in malignant glioma model mice, upregulates NK cells, and reduces Treg cells. Additionally, the expression levels of Keap1, HO-1, and Nrf2 proteins are elevated, suggesting that LBP may enhance anti-tumor efficacy by activating the Keap1/Nrf2 pathway and boosting immune responses (61). Ferroptosis is a novel form of regulated cell death, driven by iron-dependent lipid peroxidation, and is closely linked to tumorigenesis (62). A study has reported that LBP (above 4.0 mg/mL) could inhibit the survival and proliferation of human breast cancer cell lines MCF-7 and MDA-MB-231. In MCF-7 and MDA-MB-231 cells, LBP upregulated the expression of p21 while downregulating Cyclin E, indicating that LBP induces cell cycle arrest at the G0/G1 phase. Following LBP exposure, oxidized iron levels increased and reduced iron levels decreased in both MCF-7 and MDA-MB-231 cells. Moreover, the results demonstrated that the ferroptosis inhibitor Fer-1 restored cell viability, attenuated MDA and glutathione (GSH) depletion, and reduced the accumulation of intracellular labile iron in both cell lines. However, these effects were counteracted by LBP. In MCF-7 cells, protein levels of cystine transporter solute carrier family 7 member 11 (SLC7A11, also called xCT) and GPX4 were elevated; however, the addition of the xCT inhibitor (Erastin) or the GPX4 inhibitor (RSL3), in combination with LBP co-culture, led to reduced expression of xCT and GPX4, respectively. Co-treatment with LBP and these inhibitors further diminished xCT and GPX4 protein levels. These results indicated that LBP induces ferroptosis via the xCT/glutathione peroxidase 4 (GPX4) signaling pathway, thereby reducing the viability and proliferation of breast cancer cells (63) (**Figure 4**).

### 4.2 Inflammatory related diseases

Inflammation is a defensive response of the immune system to infection or tissue injury, characterized by the recruitment of immune cells and the release of inflammatory mediators to eliminate pathogens and initiate tissue repair. The immune system detects danger signals through pattern recognition receptors (such as TLRs), coordinating innate and adaptive immune responses to regulate the magnitude of inflammation (64). A study has demonstrated that Tregs modulate various aspects of monocyte behavior, including their survival, migration/infiltration, and differentiation into M2 macrophages. These regulatory functions are thought to depend on specific Treg-associated transcriptional programs and signaling pathways. Complex crosstalk between Tregs and macrophages may involve the release or expression of immunomodulatory cytokines, such as IL-10, IL-13, and IL-4, or the exchange of extracellular vesicles. Such intricate interactions can result in enhanced macrophage phagocytic activity, the release of pro-inflammatory mediators, and even the promotion of inflammatory disease pathogenesis (65). Intriguingly, microbiota-regulated immune functions have also been implicated in the development of inflammatory disorders. Approximately 70% to 80% of immune cells reside within the mesenteric lymph nodes, comprising key populations such as dendritic cells, macrophages, neutrophils, natural killer cells, and mast cells. Among these, macrophages are distributed throughout the intestinal tract, where their immunoregulatory roles are profoundly influenced by the gut microbiota. This dynamic interplay endows macrophages with pivotal functions in the modulation of immune homeostasis and the pathogenesis of inflammatory diseases (66).

In a study on DSS-induced colitis in rats, LBP (*i.g.*, 100 mg/kg) and/or capsaicin (CP) improved the disease activity index (DAI), reduced colonic injury severity, and attenuated colon shortening. Both agents, whether used alone or in combination, decreased the levels of pro-inflammatory cytokines such as IFN-γ, IL-17A, and IL-22 in colonic tissues (67). Microbiota analysis revealed that LBP and capsaicin increased the abundance of beneficial gut bacteria such as *Ruminiclostridium_9* and *Ruminoclostridium_1*, and modulated the diversity and composition of the colonic microbiota. These findings suggest that LBP and capsaicin alleviate DSS-induced colitis by suppressing inflammatory responses and modulating the gut microbiota (67). Another study demonstrated that the ameliorative effects of LBP (*i.g.*, 100 mg/kg) on DSS-induced UC model rats were associated with reduced serum MDA levels, increased catalase (CAT) activity, and decreased levels of TNF-α and IL-6 in colonic tissues (68). Similarly, LBP exerts protective effects against UC and modulates the intestinal microbiota. Specifically, taking a 1% dietary supplement of LBP could increase body weight and colon length in UC model mice while reducing disease activity scores and myeloperoxidase (MPO) activity. 16S rRNA sequencing revealed that LBP increased the abundance of probiotic genera such as Lactobacillus, unclassified *Lachnospiraceae*, *Butyricicoccus*, and *unclassified S24-7*. Moreover, LBP markedly elevated the levels of butyrate in the feces of UC mice. These results suggest that LBP alleviates intestinal inflammation by modulating the gut microbiota composition and promoting the production of microbial metabolites such as butyrate in UC mice (69). Interestingly, researchers found that a confined environment induced colonic tissue damage and inflammatory cell infiltration in mice, whereas intervention with *Lycii Fructus* water-soluble polysaccharide (LFWP) (*i.g.*, 50, 100 and 200 mg/kg) reduced serum levels of TNF-α, IL-1β, and IL-6 while increasing IL-10 levels in mice. LFWP also decreased colonic levels of LPS, TNF-α, IL-1β, and IL-6, while increasing IL-10 expression, leading to normalization of calprotectin levels and MPO activity in colonic tissues (70).

LBP has demonstrated beneficial effects in improving HE model mice. It attenuates liver injury, oxidative stress, and apoptosis, and reduces levels of pro-inflammatory cytokines TNF-α and IL-6, thereby alleviating ammonia-induced brain damage and motor dysfunction. Further investigations revealed that LBP mitigates inflammation in both the liver and brain by inhibiting the MAPK signaling pathway, effectively restoring behavioral functions in mice. In the cellular study, LBP also protected microglial cells from ammonia- or cytokine-induced damage, suggesting its potential to improve neurological function (54).

The combination of LBP and aerobic exercise (AE) improves high-fat diet-induced non-alcoholic steatohepatitis (NASH). Both LBP (*i.g.*, 100 mg/kg) and AE alone alleviated NASH symptoms, but their combined application produced more pronounced therapeutic effects (71). The synergistic treatment activated the AMPK/PPARα/peroxisome proliferator-activated receptor-gamma coactivator-1alpha (PGC-1α) signaling pathway, reducing hepatic lipid accumulation and improving serum lipid profiles and inflammatory markers in NASH model rats. Specifically, it enhanced fatty acid oxidation (FAO) and suppressed the expression of lipogenesis-related genes such as acetyl-CoA carboxylase (ACC), fatty acid synthase (FASN), and sterol regulatory element-binding protein 1c (SREBP1c). Furthermore, the combination enhanced hepatic FAO and reduced liver lipid deposition by modulating gene expression (71). Another study reported that LBP (*i.g.*, 10 mg/kg) intervention reduced liver wet weight in non-alcoholic fatty liver disease (NAFLD) rats, lowered mRNA expression levels of fibrosis markers collagen 1 (Col-I), Col-III, and α-SMA, and decreased the expression of inflammatory cytokines IL-1β, TGF-β1, and IL-6 in serum. These findings suggest that LBP alleviates hepatic fibrosis and inflammation in NAFLD rats (72).

The study has shown that the diabetic nephropathy could induced renal dysfunction, glomerular structural damage, and inflammatory infiltration in rats. Administration of LBP (*i.g.*, 100 mg/kg) alleviated renal injury, suppressed inflammation, improved kidney function, and downregulated the expression of TLR4, MyD88, and NF-κB proteins in diabetic nephropathy rats. The protective effect of LBP against diabetic renal injury is probably associated with the inhibition of TLR4/MyD88-mediated inflammatory signaling (73). LBP also exhibits protective effects in rats with chronic renal failure (CRF). Following LBP (*i.g.*, 250 mg/kg) intervention, the levels of TNF-α, IL-6, and IL-1β in renal tissues were reduced, and kidney morphology was markedly restored compared to the CRF model rats. These results suggest that LBP improves renal pathology in CRF rats, possibly by suppressing inflammatory responses (74). LBP is effective in ameliorating inflammation and oxidative stress in mice with acute pancreatitis (AP). Treatment with LBP (*i.p.*, 5 mg/mL) resulted in a reduced pancreatic index, lower histopathological scores, decreased serum levels of IL-6, IL-1β, and TNF-α, reduced MDA and MPO levels in pancreatic tissue, increased SOD levels, and downregulation of p65 mRNA and protein expression in AP model mice. These findings indicate that LBP protects against pancreatic injury in AP model mice, likely through inhibition of pro-inflammatory cytokines, oxidative stress mediators, and the NF-κB signaling pathway (75).

### 4.3 Infectious diseases

The onset and progression of infectious diseases are closely associated with impaired immune defense mechanisms. During a normal immune response, APCs degrade pathogens and present their antigenic epitopes. Subsequently, APCs display these epitopes to B cells and T cells, thereby initiating the production of cytokines and antibodies, or activating cytotoxic T cells. However, in infection-mediated autoimmunity, self-reactive T cells and/or B cells may be activated either by the presentation of self-antigenic epitopes through APCs or via bystander activation mechanisms. Once activated, these autoreactive cells can instigate autoimmune responses by generating a storm of pro-inflammatory cytokines or producing pathogenic autoantibodies that target host cells and tissues. Ultimately, this dysregulated immune activation culminates in the development of autoimmune pathology (76).

Sepsis model induction often leads to liver injury. After two weeks of LBP (*i.g.*, 400 mg/kg) treatment, serum liver enzyme and inflammatory cytokine levels were elevated, while hepatic histopathological damage was markedly alleviated compared to the septic model mice. Additionally, the anti-apoptotic protein Bcl-2 was markedly upregulated, whereas cleaved caspase-3, TLR4, and NF-κB protein levels were downregulated in liver tissue. These findings suggest that LBP attenuates inflammatory responses, liver injury, and apoptosis in septic model mice, likely via inhibition of the TLR4/NF-κB signaling pathway (77). LBP exhibits pronounced therapeutic effects on splenic immune function and prognosis in cecal ligation and puncture-induced sepsis. A study has shown that LBP (*i.g.*, 200, 400, and 800 mg/kg) improves splenic immune responses, reduces systemic inflammation, and increases the proportions of T cell subsets (CD3+, CD4+, and CD8+), thereby enhancing survival in septic rats. Moreover, LBP ameliorated splenic tissue injury, boosted immune responses, and markedly upregulated Human leukocyte antigen-DR (HLA-DR) protein expression in the spleen (10). As an adjuvant, LBP enhances the immune protective efficacy of recombinant *Lactobacillus* vaccines against Trichinella spiralis infection. Experiments showed that co-administration of LBP (*i.g.*, 100 mg/kg) and recombinant *Lactobacillus* increased levels of specific IgG, IgG1, and IgG2a antibodies, as well as sIgA levels in intestinal and muscular tissues in *T.spiralis* challenge mice. LBP also enhanced the expression of IFN-γ and IL-4 in immune cells and promoted a mixed Th1/Th2 T cell response (78). A study has reported that LBP administration effectively improves general condition and survival in immunocompromised mice with Klebsiella pneumoniae-induced pneumonia (79). LBP (*i.g.*, 40, 20, and 10 mg/kg) reduced the pulmonary index and bacterial load in lung homogenates, preserved alveolar architecture, and mitigated congestion and inflammatory exudates. Treatment also elevated total lymphocyte counts, total T cells, helper T cells, and B cells in the mice. LBP administration also increased neutrophil phagocytic index and lymphocyte proliferation, along with restoration of MPO and SOD activities in pneumonia model mice, indicating its immunostimulatory and anti-infective properties. Furthermore, LBP downregulated the abnormally elevated levels of inflammatory cytokines IFN-γ, TNF-α, and IL-6 in a dose-dependent manner (79). Interestingly, LBP exhibits inhibitory activity against various SARS-CoV-2 variants, with the study showing effective blockade of viral entry into host cells, particularly against the Omicron variant (80). LBP (50, 100, 200 or 400 μg/mL) prevents viral attachment and membrane fusion by interfering with the binding between the SARS-CoV-2 spike protein and the angiotensin-converting enzyme 2 (ACE2) receptor in 293T cells overexpressing ACE2. Experimental data suggest that LBP acts during the early stages of viral invasion, and intranasal administration of LBP (1.6, 3.2 and 6.4 mg/mL) reduced pseudovirus entry and alleviated nasal mucosal damage caused by Omicron in Omicron PsV mice (80). Surface plasmon resonance assays revealed dual binding of LBP to both the SARS-CoV-2 spike protein and the ACE2 receptor, further confirming its inhibitory effect via disruption of the spike–ACE2 interaction. These findings indicate that LBP may serve as a broad-spectrum viral entry inhibitor with potential therapeutic value against SARS-CoV-2 (80) (**Table 2**).

**Table 2:** Research on LBP in immune-related diseases.

Animals or cells	Disease models	Dosage of administration	Administration time	Mechanisms	Outcomes	References
Female BALB/c mice	Breast cancer	Intratumoral injection, 2 mg/mL	15 days	Activated innate immune responses in macrophages via the TLR4-myd88-IKK-NF-κB signaling pathway and upregulated the expression of immune-related genes	Suppressed tumor growth	(59)
Female C57BL/6 mice	Malignant glioma	*i.g.*, 50 mg/kg	4 weeks	Activated the Keap1/Nrf2 pathway and boosted immune responses	The tumor morphology tended to be normal	(61)
Human breast cancer cell lines MCF-7 and MDA-MB-231	Breast cancer	Above 4.0 mg/mL	48 h	Induced ferroptosis via the xCT/GPX4 signaling pathway, thereby reducing the viability and proliferation of breast cancer cells	Reduced the survival and proliferation of breast cancer cells	(63)
Male SD rats	Colitis	*i.g*., 100 mg/kg	4 weeks	Decreased the levels of pro-inflammatory cytokines such as IFN-γ, IL-17A, and IL-22 in colonic tissues, and increased the abundance of beneficial gut bacteria such as *Ruminiclostridium_9* and *Ruminoclostridium_1*, and modulated the diversity and composition of the colonic microbiota	Colon length increased and Weight/Length ratio decreased	(67)
Male kunming mice	Colonic tissue damage and inflammation	*i.g*., 50, 100 or 200 mg/kg	15 days	Decreased colonic levels of LPS, TNF-α, IL-1β, and IL-6, while increasing IL-10 expression, leading to normalization of calprotectin levels and MPO activity in colonic tissues	Improved colonic ulceration and inflammatory cell infiltration	(70)
Male SD rats	Non-alcoholic steatohepatitis	*i.g*., 100 mg/kg	10 weeks	Enhanced FAO and suppressed the expression of lipogenesis-related genes such as ACC, FASN, and SREBP1c	Alleviated hepatocyte injury, and reduced body weight, serum lipid and inflammation levels	(71)
SD rats	Non-alcoholic fatty liver disease	*i.g*., 10 mg/kg	8 weeks	Lowered mRNA expression levels of fibrosis markers Col-I, Col-III, and α-SMA, and decreased the expression of inflammatory cytokines IL-1β, TGF-β1, and IL-6 in serum	Reduced liver wet weight and liver fibrosis	(72)
Male SD rats	Diabetic nephropathy	*i.g*., 100 mg/kg	6 weeks	Inhibited TLR4/myd88-mediated inflammatory signaling	Ameliorated the damage of glomerular epithelial cells in diabetic kidney injury rats	(73)
Male SD rats	Chronic renal failure	*i.g*., 250 mg/kg	28 days	Reduced the levels of TNF-α, IL-6, and IL-1β in renal tissues	Improved glomerular atrophy, renal tubular epithelial cell edema, inflammatory cell infiltration	(74)
Male kunming mice	Acute pancreatitis	*i.p.*, 5 mg/mL	At 0 h and 4 h	Inhibited the pro-inflammatory cytokines, oxidative stress mediators, and the NF-κB signaling pathway	The pancreas coefficient and pancreatic histopathological score decreased	(75)
Male BALB/c mice	Sepsis	*i.g.*, 400 mg/kg	2 weeks	Attenuated inflammatory responses, liver injury, and apoptosis via inhibition of the TLR4/NF-κB signaling pathway	Improved liver injury caused by sepsis	(77)
Male C57BL/6j Gpt and K18-hACE2 mice	SARS-coV-2	1.6, 3.2 or 6.4 mg/mL	3 days	Dual binding of LBP to both the SARS-coV-2 spike protein and the ACE2 receptor, confirming its inhibitory effect via disruption of the spike-ACE2 interaction	It effectively inhibited the early infection of the virus	(80)

## 5 Application prospects of LBP in functional foods

Functional foods not only provide basic nutrition but also offer specific health benefits, such as immune enhancement, anti-aging, antioxidant activity, and metabolic regulation. As a natural, non-genetically modified bioactive compound with multiple health-promoting properties, LBP is increasingly being incorporated into functional food products, particularly for its potential in boosting immunity, combating aging, and regulating metabolism.

LBP serves as a key component in immune-enhancing functional foods, with its immunomodulatory effects well-documented through extensive research. It enhances immune responses by promoting the proliferation of macrophages, T cells, and B cells, thereby improving immune cell function (4, 11). Consequently, LBP is widely used in foods aimed at supporting immune health. LBP also holds great promise in elderly-targeted nutrition, particularly due to its dual effects on gut microbiota and immune enhancement. Various pre-treatment methods combined with mild acid extraction have been used to obtain LBP with distinct physicochemical properties and bioactivities. Notably, experiment results found that LBP obtained via a combination of mild acid extraction and enzymatic pre-treatment (e.g., S1-M1) promotes the growth of beneficial gut bacteria (e.g., *Bacteroides*) and enhances immune system function. Additionally, LBP exhibits excellent thickening properties, making it suitable for use in foods designed for elderly individuals with dysphagia (81). LBP also exerts neuroprotective effects by modulating immune function, a property that proves particularly advantageous in the context of neurodegenerative diseases such as Alzheimer’s disease (AD) and Parkinson’s disease (PD). Through its immunoregulatory actions, LBP may attenuate neuroinflammation, mitigate neuronal damage, and thereby contribute to the prevention or amelioration of neurodegenerative processes associated with these disorders (**Table 3**). This study offers a strategic basis for developing functional foods targeting gut dysbiosis and immune senescence.

**Table 3:** Research on LBP in neurological conditions.

Animals or cells	Disease models	Dosage of administration	Administration time	Mechanisms	References
SD rats	AD model	*i.g.*, 300 mg/kg	1 week	Reduced the levels of IL-4, IL-10, NF-κB, TNF-α, and IL-1β in the cerebral cortex	(98)
Silkworm	PD model	Medicaments for feeding, 50 or 100 mg/kg	5 days	Increased dopamine levels, upregulate tyrosine hydroxylase protein expression, and restored dopa decarboxylase mRNA expression levels	(99)
Male C57BL/6J mice	Peripheral nerve injury (PNI) and optic nerve crush (ONC) model	*i.g.*, 100 mg/kg or intravitreal injection 10 µg/µl	ONC: 21 days (*i.g.*) and twice (intravitreal injection) PNI: 21 days or 28 days	Enhanced the intrinsic growth capacity and functional recovery of adult neurons following PNI, induced axonal regeneration within the central nervous system, and promoted the survival of retinal ganglion cells	(92)
Female SD rats	Ocular hypertension model	*i.g.*, 1 mg/kg	28 days	Reduced the loss of retinal ganglion cells	(100)
SD rats	Ocular hypertensive model	*i.g.*, 1 mg/kg	35 days	Restoration of the photopic negative response in the superior retina to normative levels mitigates the functional impairment caused by partial optic nerve transection.	(101)
PC12 cells	6-OHDA treatment	100-600 μg/mL	24 h	Attenuation of intracellular ROS and NO accumulation, reduction of 3-nitrotyrosine and intracellular free Ca²^+^ levels, and inhibition of excessive expression of NF-κB, nNOS, and iNOS	(102)
PC12 cells	H_2_O_2_ treatment	125, 250 or 500 μg/mL	24 h	Inhibited mitochondrial apoptosis and activate the cellular Nrf2/HO-1 signaling pathway	(103)
Male C57BL/6 mice	AD model	*i.g.*, 50, 100 or 200 mg/kg	28 days	Modulated the expression of proteins associated with the insulin receptor substrate-1 (IRS1)/PI3K/Akt signaling pathway, thereby reducing Aβ deposition and tau protein phosphorylation	(104)
Male C57BL/6 mice	PD model	*i.p*., 100 or 200 mg/kg	16 days	Elevated the expression levels of SOD2, CAT, and GPX1, suppressed the MPTP-induced aberrant aggregation of α-synuclein, and activated the phosphatase and tensin homolog (PTEN)/Akt/mTOR signaling pathway	(105)

LBP could serve as a principal component in anti-aging functional foods. Aging is an inevitable physiological process, during which oxidative stress and inflammatory responses play critical roles (82). LBP has been shown to enhance immune function and effectively delay aging. Fermented *Lycium barbarum* polysaccharide (FLBP) exhibits protective effects on the intestines of aging model mice. FLBP (*i.g.*, 150 and 300 mg/kg) counteracts D-galactose-induced growth retardation and thymic atrophy in mice, decreases serum levels of TNF-α, IL-1β, and IL-6, and increases IL-10 levels. It also improves colonic barrier integrity, as indicated by reduced intestinal permeability and serum LPS and LBP levels, altered colonic tissue morphology, and increased mucin 2 secretion (83). FLBP also modulates gut microbiota composition by altering alpha and beta diversity, increasing beneficial taxa such as *norank_f_Muribaculaceae* and *Lactobacillus*, reducing harmful genera like *Atopostipes* and *Jeotgalicoccus*, and elevating fecal levels of SCFAs including acetate, propionate, valerate, and isobutyrate. These findings suggest that FLBP’s anti-inflammatory effects and ability to reduce serum proinflammatory cytokines are linked to improved colonic barrier function, modulation of gut microbiota, and increased levels of beneficial SCFAs (83). Thus, LBP represents a valuable ingredient in anti-aging functional foods, with considerable market potential.

LBP could be utilized as a supplementary component in metabolism-regulating functional foods. The study has shown that high-fat diets impair growth, reduce hepatic antioxidant capacity, and disrupt lipid metabolism in Cyprinus carpio. Dietary supplementation with 0.5-2.0 g/kg of LBP improved growth performance, increased total hepatic antioxidant capacity, enhanced CAT and GSH-Px activities, and reduced MDA content. LBP also modulated lipogenic enzyme activity, reduced fat deposition, and decreased hepatic lipid accumulation by regulating the expression of lipid metabolism-related genes (84). In mice, LBP supplementation (150 mg/kg) reduced serum and hepatic total cholesterol (TC), triglycerides (TG), and free fatty acids in high-fat diet-induced obese models. Gene expression analysis revealed that LBP feeding upregulated adiponectin and downregulated lipogenesis-related genes such as PPARγ, CD36, ACC, and FASN. Moreover, LBP intake altered the gut microbiota composition in mice by increasing *Bacteroidetes* and decreasing *Firmicutes* abundance, indicating its potential to modulate the gut microbiome (85). Therefore, LBP holds significant potential in the prevention and management of lipid metabolism disorders and could serve as an adjunctive therapeutic agent for chronic conditions such as obesity.

As an additive in gut-friendly probiotic foods, LBP primarily exert their effects through prebiotic activity, immunostimulatory properties, and modulation of the gut microbiota. The study has shown that LBP solution (0.1 mL/10 g) promotes the growth of beneficial bacteria such as *Lactobacillus* and *Bifidobacterium*, and increases the abundance of protective microbes like *Akkermansia*, *Lactobacillus*, and *Prevotellaceae* by altering the composition of the gut microbiota in mice. LBP also enhances the innate immune response in mice, significantly increasing serum levels of TGF-β and IL-6, as well as sIgA levels in colonic contents (26). High-throughput 16S rRNA sequencing revealed that LBP intake promotes microbial diversity in the gut and alters the microbial composition, notably increasing the relative abundance of *Firmicutes* and *Proteobacteria* (26). Another study reported that combining LBP with AE significantly alleviated hepatic lipid accumulation, dyslipidemia, and gut microbiota imbalance in NAFLD mice. The combined treatment of LBP (*i.g.*, 50 mg/kg added in diet) and AE restored intestinal barrier function, elevated beneficial SCFA levels, modulated gut microbial composition, and reduced hepatic inflammatory responses. Furthermore, the LBP + AE combination had a more pronounced effect on gut microbial diversity than either treatment alone, particularly by increasing beneficial *Butyricicoccus* and *Butyricimonas* populations and decreasing harmful *Proteobacteria* (86). Interestingly, LBP has also been shown to enhance immunity, antioxidant capacity, and gut microbiota modulation in *Luciobarbus capito*. Experimental findings demonstrated that 0.10 g/L LBP culture solution increased hepatic antioxidant enzyme activity, intestinal digestive enzyme activity, and immune enzyme levels in *L.capito*. 16S rRNA gene sequencing analysis revealed that LBP administration altered the gut microbiota composition of *L.capito*, particularly affecting *Proteobacteria* and *Firmicutes* populations, indicating a positive impact on microbial metabolic functions that may enhance the fish’s immune defense and disease resistance (87). Therefore, LBP has broad market potential for application in gut-health functional foods, particularly as a component of prebiotic products. By modulating the gut microbiota and enhancing intestinal barrier function, LBP offers novel strategies for the prevention and treatment of gastrointestinal disorders.

Although LBP exhibits considerable health-promoting properties as a functional food ingredient, significant challenges persist in quality control. Foremost among these is the standardization of LBP content in functional foods. Variations in *Lycium barbarum* cultivars, geographic origin, harvesting season, and processing methods lead to substantial fluctuations in polysaccharide content, complicating the establishment of uniform benchmarks. Such variability may also introduce inconsistencies during polysaccharide extraction. Current analytical methods for LBP quantification, such as the phenol–sulfuric acid assay, are susceptible to interference from other saccharides and lack specificity, thereby affecting the comparability of results. Comprehensive analysis of LBP in experimental settings is complex, underscoring the need for a simplified and scalable industrial detection method (88, 89). Furthermore, regulatory frameworks must strike a balance between scientific rigor and practical operability. It is imperative to establish graded standards based on molecular weight distribution and bioactivity indices (e.g., immunomodulatory effects), implement traceability management for raw *Lycium barbarum* materials, require the declaration of LBP content ranges in product labeling, and develop standardized criteria for LBP-enriched functional foods.

## 6 Limitations of LBP research

Although LBP have demonstrated promising application potential in research, several limitations hinder their broader practical use.

First, there are limitations in the extraction and purification techniques of LBP. These are critical to its application, yet traditional methods often damage the polysaccharide structure, thereby reducing its bioactivity (90). Different extraction methods influence the structure of LBP. For instance, low-temperature alkaline extraction (0.6% NaOH) preserves more of the rhamnogalacturonan I (RG-I) regions, whereas high-temperature acid extraction (0.4% HCl) retains more homogalacturonan (HG) regions but results in substantial degradation of side chains, such as arabinose. Hot water extraction yields acidic heteropolysaccharides that are larger and exhibit a highly branched structure. Structural analyses further confirmed these differences, demonstrating that extraction methods distinctly affect the molecular morphology and degree of branching in LBP (91). Therefore, future research should focus on optimizing LBP extraction techniques to develop methods that preserve or enhance immunological bioactivity. Meanwhile, we have noted an intriguing study in which the researchers explored the mechanism of LBP through a bioinformatics approach involving the comparison with currently Food and Drug Administration (FDA)-approved drugs. Specifically, they first identified the targets modulated by LBP, and then constructed a bioinformatics database of FDA-approved drugs to screen for compounds with overlapping targets—particularly those drugs whose mechanisms of action and target profiles closely resemble those of LBP. Finally, the mechanisms underlying these candidate drugs were investigated in detail (92). This research paradigm may provide valuable insights for the effective screening and functional characterization of LBP.

Secondly, the bioavailability and absorption mechanisms of LBP remain poorly understood. Although LBP exhibits strong biological activity in experimental models, its unclear bioavailability represents a significant barrier to clinical application. Current evidence suggests that LBP is poorly absorbed in the gastrointestinal tract and exhibits low stability during digestion, which may contribute to its limited bioavailability (93). To enhance bioavailability, researchers have explored various strategies, such as encapsulating LBP in nanoliposomes to improve its immunomodulatory effects (94). Although nanotechnology has shown promise in enhancing LBP’s bioactivity, its long-term effects and safety require further investigation. Consequently, deeper exploration into LBP’s absorption mechanisms and strategies for improving its bioavailability is essential for its successful clinical use.

Lastly, there is a lack of clinical data and practical application. Most LBP research remains limited to animal and *in vitro* study, with a notable absence of large-scale clinical trial data. Some studies have explored LBP as a potential prebiotic for improving gut dysbiosis in patients with NAFLD (95), as an adjuvant therapy for type 2 diabetes (96), and as a means of alleviating depressive symptoms in adolescents with subthreshold depression (97). Although preliminary clinical findings support LBP’s notable bioactivity, existing trials are insufficient to fully establish its clinical safety and efficacy. Therefore, future efforts should prioritize rigorous clinical research, especially large-scale, multicenter randomized controlled trials (RCTs), to comprehensively evaluate LBP’s therapeutic effects and safety across various diseases.

## 7 Conclusion

LBP holds significant potential for modulating immune function. It could enhance immunity, regulate gut microbiota, slow aging processes, and inhibit tumor growth, offering preventive and therapeutic benefits against various immune-related diseases. However, current investigations remain largely confined to animal and *in vitro* study, and clinical applications are still in their infancy. Further research is warranted. Future studies should focus on optimizing extraction methods, elucidating underlying mechanisms, and conducting large-scale clinical trials to ensure the safety and efficacy of LBP in clinical practice.
